# Multifaceted Impact of the Coronavirus Disease 2019 (COVID-19) Pandemic on ST-Elevation Myocardial Infarction (STEMI): A Literature Review of Incidence, Treatment Modalities, and Outcomes

**DOI:** 10.7759/cureus.57288

**Published:** 2024-03-30

**Authors:** Hoi K Choi, Madhurima Maity, Mohammed Qureshi, Ali Haider, Sagar Kapadia, Sofia Fuerte, Simon Antony, Waleed Razzaq, Anum Akbar

**Affiliations:** 1 Internal Medicine, University of Michigan, Ann Arbor, USA; 2 Internal Medicine, Sir H. N. Reliance Foundation Hospital and Research Centre, Mumbai, IND; 3 Internal Medicine, American University of the Caribbean School of Medicine, Cupecoy, SXM; 4 Medicine, Quetta Institute of Medical Sciences, Quetta, PAK; 5 Emergency Medicine, Jio World Centre, Mumbai, IND; 6 Internal Medicine, Instituto Tecnológico y de Estudios Superiores de Monterrey, Monterrey, MEX; 7 Internal Medicine, Sri Ramachandra Institute of Higher Education and Research, Chennai, IND; 8 Internal Medicine, Services Hospital, Lahore, PAK; 9 Pediatrics, University of Nebraska Medical Center, Omaha, USA

**Keywords:** acute coronary syndrome, covid-19 vaccination, st-elevation myocardial infarction (stemi), myocardial infarction, covid-19

## Abstract

The global repercussions of coronavirus disease 2019 (COVID-19) include substantial worldwide mortality and have brought to light existing gaps in healthcare systems. Particularly, diseases requiring time-sensitive treatment, such as ST-elevation myocardial infarction (STEMI), have faced significant challenges due to the impact and revelations of the COVID-19 pandemic on healthcare infrastructure. This review addresses the impact of the pandemic on STEMI, exploring incidence, treatment modalities, and clinical outcomes. Through a critical examination of existing literature, the intricate relationship between the pandemic and cardiovascular health, specifically STEMI, is elucidated. The COVID-19 pandemic has had a significant impact on the management of STEMI, with changes in hospitalization rates, treatment strategies, and the presentation of the disease posing significant challenges. The contradictory results of COVID-19 and post-vaccine myocardial infarction, as well as gender differences in reported cases, highlight the need for further research to clarify these relationships.

## Introduction and background

The global impact of the coronavirus disease 2019 (COVID-19) caused by severe acute respiratory syndrome coronavirus 2 (SARS-CoV-2) has been profound, leading to millions of infections and nearly a million deaths worldwide. The pandemic significantly strained healthcare systems worldwide, particularly in resource-limited countries, affecting health service delivery, human resource management, facility utilization, and medical supply chains [1]. It not only directly impacted health systems but also exposed pre-existing gaps, disrupting both preventive and curative services for various diseases [1]. Delays in essential services, cancellations of planned treatments, and shortages of healthcare workers, medicines, and technologies contributed to these disruptions [2].

**Figure 1:**
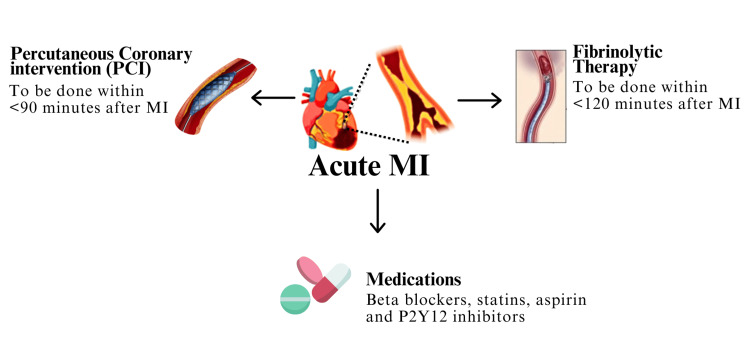
Treatment of STEMI. MI: myocardial infarction; P2Y12: purinergic receptor P2Y, G-protein coupled; STEMI: ST-elevation myocardial infarction Source: [13]

Research indicates that deficiencies in the healthcare system, coupled with the challenges of accessing healthcare during a pandemic, have had a substantial impact on admissions and care for ST-elevation myocardial infarction (STEMI) [3]. One notable consequence of the COVID-19 pandemic was a substantial decrease of around 40% in cases of STEMI compared to the pre-pandemic period [4]. STEMI, characterized by myocardial ischemia, EKG changes, and chest pain, results from coronary artery occlusion, commonly caused by plaque-related issues [5]. STEMI, a critical condition with potentially life-threatening consequences, is a manifestation of transmural myocardial ischemia [6]. STEMI commonly occurs due to acute thrombotic occlusion in the coronary artery, while plaque erosion and calcific nodules contribute to a lesser extent [7]. Primarily, risk factors for STEMI include dyslipidemia, diabetes, hypertension, smoking, and a family history of coronary artery disease [8]. Globally, over three million people every year present with STEMI, while in the United States, it affects approximately 750,000 people each year posing a significant health burden [9,10]. The possible pathophysiology of varied expressions of STEMI during the COVID-19 pandemic can be explained by the following mechanisms. After contracting SARS-CoV-2, the heart tissue may sustain damage through various mechanisms, including viral entry into cardiac cells, inflammation, oxidative stress, microvascular clot formation, and oxygen imbalance [11]. This damage can lead to myocardial injury and different types of myocardial infarction (MI). Furthermore, dysregulation of the renin-angiotensin system (RAS) is identified as a key factor in myocardial injury during COVID-19 [12]. The virus binds to angiotensin-converting enzyme 2 (ACE2), a major regulator of RAS, facilitating its entry into host tissues, including the myocardium. Once inside, the virus replicates and releases RNA, potentially leading to the destruction of host cells. Additionally, the downregulation of ACE2 by the virus can exacerbate the harmful effects of angiotensin II, contributing to endothelial dysfunction, vasoconstriction, and microthrombus formation, further damaging cardiac cells [12]. The immune response triggered by the virus can also indirectly harm the heart through hypoxemia. Treatment for STEMI (Figure 1) involves prompt intravenous access, oxygen therapy for hypoxemic patients, and either percutaneous coronary intervention (PCI) within 90 minutes or fibrinolytic therapy within 120 minutes. Medications, including beta blockers, statins, aspirin, and P2Y12 inhibitors, play a crucial role in management [13]. The pandemic's indirect effects, such as healthcare worker reassignment, treatment cancellations, and economic challenges, were among the reasons for reduced STEMI cases [14]. Despite changes in hospital procedures and increased ambulance response times due to the COVID-19 surge, there is no observed delay in achieving coronary revascularization or worsened clinical outcomes [15]. However, COVID-19 patients with STEMI tend to have a higher burden of coronary thrombus compared to non-COVID-19 patients [15]. Analysis of STEMI outcomes during the pandemic reveals significant challenges, including a threefold increase in in-hospital mortality for STEMI patients with COVID-19 and delays in receiving proper treatment [16]. Complications such as stent thrombosis and cardiogenic shock are more common, especially among older patients, who also face longer procedure times and higher mortality rates [17]. Patients with pre-existing cardiovascular conditions experience elevated mortality rates, underscoring the need for tailored management and improved healthcare access [18]. These challenges highlight the intricate relationship between the pandemic and STEMI management, emphasizing the importance of ongoing adaptation in healthcare systems to optimize patient care.

This review aims to comprehensively analyze the impact of the COVID-19 pandemic on STEMI, exploring various aspects such as incidence, treatment modalities, and clinical outcomes. By critically examining existing literature, the goal is to elucidate the intricate relationship between the pandemic and cardiovascular health, particularly in the context of STEMI. Understanding how COVID-19 affects different age groups concerning STEMI is important for risk stratification, resource allocation, and tailored interventions based on age-specific patterns of incidence, treatment responses, and outcomes. Physiological differences, comorbidities, and socioeconomic factors contribute to variations in the presentation and management of STEMI across age groups, informing clinicians about optimized patient care.

## Review

Incidence of STEMI during the COVID-19 pandemic

During the COVID-19 pandemic, variation in the incidence and prevalence of acute coronary syndrome (ACS), particularly STEMI infarction, was seen across many continents. This section aims to evaluate the incidence of STEMI by analyzing the reported cases of hospital admission during the COVID-19 pandemic in different regions of the world, such as Europe, North America, Africa, Asia, and Australia.

In North America

A study conducted in 2020 shed light on the incidence of MI in North America amidst the COVID-19 pandemic. The study found a reduction in MI cases by 19% during the pandemic. Among patients receiving primary PCI during the lockdown, there was a median increase of 44 minutes in symptom-to-balloon time [19]. Concurrently, another study reported consistent STEMI admissions during the lockdown, albeit with significant increases in time intervals between symptom onset and first medical contact (FMC) [20]. In a study conducted by Zitelny et al., it was revealed that there was a 9.5% decrease in STEMI admissions from the monthly averages of 2018 and 2019. Moreover, there was a 14.6% decrease in cases between January and March 2020, marking the first instance in two years where March recorded fewer cases than January [21]. These findings collectively highlight the impact of the pandemic on STEMI incidence and healthcare utilization.

In the United Kingdom

A study conducted by Mafham et al. documented a decline in ACS admissions from mid-February to the end of March 2020, followed by a partial reversal from April to May 2020, affecting all types of ACS, including STEMI [22]. Another study highlighted disparities in hospitalization rates for AMI among individuals from Black, Asian, and Minority Ethnic (BAME) backgrounds during the COVID-19 era, with BAME patients experiencing higher rates of hospitalization, particularly among younger males presenting with ST-elevation AMI [23]. Moreover, during the pandemic, BAME individuals faced notably higher in-hospital and seven-day mortality rates compared to the pre-pandemic era. The research findings highlight notable trends and disparities in patient care and outcomes in the United Kingdom, contributing substantially to our understanding of cardiovascular health during these challenging times.

In Europe

Correspondingly, amidst the COVID-19 pandemic, numerous studies from across Europe have also underlined the incidence and management of STEMI. A study observed a significant decline in both suspected and confirmed STEMI cases compared to the previous year, despite persistent reperfusion strategies [24]. Another study noted that individuals during the outbreak were typically older and tended to delay seeking medical care, resulting in a twofold increase in in-hospital mortality rates [25]. Similarly, Hauguel-Moreau et al. highlighted a notable threefold increase in ischemic time in STEMI management, primarily due to patient-related delays [26]. Furthermore, another study analyzed patients undergoing primary PCI for STEMI within 24 hours, revealing a significant reduction in the average number of STEMI cases per month during the lockdown period compared to pre-lockdown, alongside increased delays from symptom onset to first medical contact [27]. These findings highlight the multidimensional challenges and implications of managing STEMI during the COVID-19 pandemic in Europe.

In Asia

A study found an over 10% reduction in hospitalization due to STEMI during the COVID-19 pandemic [28]. Similarly, a study conducted in India also showed a reduction in STEMI cases during the COVID-19 pandemic [29].

In Australia

In 2020, Chan et al. conducted a study revealing a decrease in hospital admissions for ACS during the five-week lockdown period compared to the pre-lockdown period, primarily due to fewer admissions for non-STEMIs (NSTEMIs), although there was no significant change observed for STEMI [30]. Furthermore, Sutherland et al. discovered a decrease in the total number of patients presenting with ACS during the pandemic, with a higher proportion of cases involving STEMI [31]. 

In Africa

Studies conducted in South Africa indicated a slight decrease in hospital admission rates for ACS during the pandemic. In March 2020 compared to the previous year, although admissions for NSTEMI showed a slight increase, no significant change was observed in admissions for STEMI [32]. Following the implementation of full lockdown measures, the most substantial change in ACS admissions was observed for unstable angina, with a 43% reduction attributed to adverse changes in the magnitude of MIs, unspecified, NSTEMI, and STEMI admissions. Similarly, another study reported a significant drop in ACS admissions during the pandemic compared to the prior year, with the lowest point representing a 46% reduction [33].

While an increased number of hospitalizations often suggests a higher prevalence of severe cases, it does not provide a comprehensive view of the overall disease incidence. Numerous factors during the COVID-19 pandemic could influence hospitalization rates. Concerns about exposure, limited transportation options, insufficient insurance coverage, and financial constraints for treatment and hospitalization are among the considerations. This necessitates a comprehensive assessment of epidemiological indicators, including confirmed case counts, mortality rates, and positivity rates, to acquire a comprehensive understanding of the disease's overall impact. To precisely gauge disease incidence, it is essential to factor in contextual elements and employ a blend of data sources and surveillance methods. Relying solely on indicators like decreased hospitalization rates, extended time intervals to medical contact, and variations in ACS admissions in different regions poses challenges for an accurate assessment of myocardial STEMI incidence during the pandemic. The mentioned challenges encompass the potential underreporting of STEMI cases, as milder instances might not lead to hospitalization, thus skewing the perceived incidence. Moreover, prolonged time intervals to medical contact can obscure the true timeline of symptom onset to healthcare interaction, impacting the reliability of these indicators. Fluctuations in ACS admissions across diverse regions further complicate the assessment, making it difficult to ascertain whether changes in hospitalization rates are solely reflective of STEMI incidence or influenced by broader healthcare system dynamics. These challenges underscore the need for a more nuanced and comprehensive approach when assessing the true incidence of STEMI during the COVID-19 pandemic. This highlights the importance of a comprehensive approach that considers diverse contextual factors influencing healthcare-seeking behavior and reporting practices in the evaluation of disease incidence.

Treatment, outcome, and prognosis of STEMI during the COVID-19 pandemic

Treatment Strategies

The pandemic was one of the greatest crises faced by modern medicine, and every advancement made until then hit a setback in the face of such adversity. Time-sensitive treatments in acute conditions like STEMI were especially challenging with the healthcare system crumbling under the effects of COVID-19.

The pre-pandemic era witnessed PCI emerge as one of the best treatment modalities for STEMI patients if door-to-balloon (D2B) time could be maintained <90 minutes. During the early stages of the pandemic, there was an estimated 40% reduction in PCI, an optimal treatment for STEMI, performed in patients [34]. The total number of hospitalized patients declined by about 26% per week nationally and by about 62% in Hubei province [34]. With major lockdowns in place and hospitals facing the acute burden of rapidly deteriorating patients, triaging emergent and non-emergent was key to optimizing resource allocation. This discouraged symptomatic STEMI patients from seeking medical care in time. The logistical difficulties with lockdowns further fuelled the delays. A significant increase in D2B time was seen which contributed to lower health quality indicator (HQI) in STEMI patients [35].

It was difficult to maintain the revascularization time targets (<120 minutes) for STEMI patients, thus increasing total ischemic time and leading to poor prognosis even post-interventions. Many STEMI patients with concurrent COVID-19 had an atypical symptomatic presentation with the absence of blockages in angiographies. This contributed to delayed interventions and higher mortality in such patients [36].

Once STEMI patients reached hospitals, clinicians struggled to screen patients for COVID-19 and minimize their nosocomial contamination with personal protective equipment (PPEs) and other protective measures to prevent the spread of the virus, thus compounding treatment delays. Fibrinolytic therapies in such situations became a favorable choice for the treatment of pandemic STEMIs [37].

During the COVID-19 pandemic, a significant shift from PCIs to fibrinolytic therapy was noted worldwide with reperfusion rates as high as 88.9%. This treatment regime included dual antiplatelet therapy along with boluses of loading doses of heparin. The re-adjusted management strategy for pandemic STEMI patients proved to be clinically acceptable after adjusting compounding factors between pre-COVID-19 and COVID-19 times [38].

Ethnic minorities (Asians, Blacks, Hispanics) had higher hospitalization and lower revascularization rates with many not undergoing PCI compared to Whites as per a national study conducted in the United Kingdom during the pandemic. Women among them had even lower odds of receiving invasive treatments than men [39].

Even though elective coronary artery bypass grafts (CABGs) might seem to increase infection risks during a pandemic, if performed safely undertaking all protective measures as per infection control guidelines, they have been shown to have acceptable complications and mortality rates [40].

A new treatment modality that was found to be quite useful in effectively treating patients while minimizing contamination of patients as well as healthcare professionals was the robotic-assisted PCI (R-PCI). Compared to the standard PCI (S-PCI), RPCI allowed the remote operation of devices, significantly decreasing person-to-person contact during the pandemic while also reducing radiation exposure. A longer procedural time (>10 minutes) was noted compared to S-PCI, but during virus outbreaks, infection control while delivering effective treatment becomes a priority above all [41].

A guideline for acute MI patients was released in the wake of the COVID-19 outbreak by the Chest Pain Center Committee, Medical Quality Control Center of Cardiovascular Diseases, Liaoning, China, which stated that patients with chest pain should be screened and triaged into four groups: (a) confirmed, (b) suspected, (c) ruled out, and (4) cannot be ruled out at present. STEMI patients, after the abovementioned classification, were further divided based on symptom onset times to aid decision-making on treatment modality (thrombolysis vs. PCI). This guide was found to be useful in the efficient allocation of the limited available resources like isolation wards and ICUs, thus helping sicker patients avail more intensive care [42].

The period of the COVID-19 pandemic provides an opportunity for contemplation on current STEMI treatment methods and the impact of management under resource constraints. Comparable circumstances may arise in numerous impoverished developing nations, both before and after the pandemic. Strategies need revision to address future crises, ensuring resilience during challenging times without compromising the care of STEMI patients.

Outcome and Prognosis

In analyzing the findings of individuals who have had STEMI against the difficult backdrop of the current pandemic, it is essential to investigate the complicated elements of death rates, comorbidities, and long-term effects.

According to the Centers for Disease Control and Prevention (CDC), there is an increase of around 50% in mortality (due to all causes) compared to pre-pandemic periods in the United States [43]. A noteworthy trend in mortality rates among STEMI patients during the pandemic has been observed. A study has shown that there has been a threefold increase in in-hospital mortality for STEMI patients with COVID-19 [16]. Besides, during the pandemic, STEMI patients have been found to have a higher need for circulatory support, worse thrombolysis in myocardial infarction (TIMI) flow, and a significantly higher 30-day mortality rate [1]. Numerous factors may contribute to the poor progress and outcomes in STEMI cases during the COVID-19 pandemic. Some of those factors include delays in receiving suitable treatment, arising from patient apprehension about visiting healthcare facilities or resource inadequacies in hospitals and clinics. Additionally, disruptions in standard management protocols and the presentation of STEMI patients with concurrent COVID-19 infections can further impact overall outcomes [44,45]. In-hospital mortality and heart failure showed a rising trend in successfully treated STEMI patients during the outbreak possibly due to delayed reperfusion by almost 20 minutes on average [46]. A study has shown that patients with STEMI and COVID-19 had higher hospital charges and a longer time from admission to PCI compared to those without COVID-19 [47]. Several studies have also reported poorer outcomes for STEMI patients with a concomitant COVID-19 diagnosis compared to historical control patients, including higher mortality rates, longer length of stay, and an increased risk of stent thrombosis [44]. Post-PCI, STEMI patients with COVID-19 developed significant stent thrombosis and cardiogenic shock compared to their non-COVID-19 counterparts. Additionally, more than half of COVID-19 patients with STEMI may develop different stages of heart failure, and an increased incidence of cardiac arrest has been observed in these patients [48]. Patients presenting with heart failures were more along with COVID-19 STEMI patients and required a higher degree of aggressive antithrombotic management to prevent acute stent thrombosis [49]. Stent thrombosis, a fatal complication of PCIs, was notably higher (up to 10 times) in infected and even COVID-19-vaccinated patients due to probable increased hypercoagulable state from the pathogen [50]. Cardiogenic shock is also more prevalent in COVID-19 patients with STEMI. COVID-19 is an independent risk factor for poor prognosis in patients with MI, leading to higher in-hospital mortality, increased risks of long-term cardiovascular events, and a higher cardiovascular burden [51]. The prevalence of various cardiac and non-cardiac complications, morbidity, and mortality among hospitalized STEMI patients with concurrent COVID-19 infection remains poorly defined [51]. Furthermore, a study has shown that ACE function is disrupted by a viral infection, leads to disturbance in RAS, and results in cardiovascular complications such as hypertension, endothelial dysfunction, and vascular inflammation [52,53].

The impact of the pandemic on STEMI patients' outcomes varies depending on their age group. In patients with STEMI, the advanced age, >65 years, was associated with a 15% increase in FMC procedure time during COVID-19. And the FMC procedure time was even longer, i.e., 15-22%, for the patients from rural and disadvantaged areas [54]. According to a study held in Germany, during COVID-19 times, the patients who presented with STEMI had a mean age of 65 years, a significantly reduced left ventricular ejection fraction (EF), and worse thrombolysis in MI and required circulatory support more often. These patients have higher 30-day mortality [55]. Elderly patients are known to have a higher thrombotic and bleeding risk and worse periprocedural outcome, which makes them more susceptible to the deleterious direct and indirect effects of COVID-19 [56]. During the pandemic, longer ischemia time and rates of late presentation were observed in both young and elderly patients. Frailty was a noteworthy contributor to all-cause mortality in post-PCI patients. Existent higher mortality rates in aged STEMI patients were only exacerbated by the concurrent COVID-19 infection. Cognitively impaired patients were at high risk for major adverse cardiovascular events (MACE). The inability to follow personal protective measures could have only increased the risk fourfold in such patients [57]. The higher thrombotic and bleeding risk in elderly patients, coupled with worse periprocedural outcome, has been reported in previous studies [56]. Moreover, age has been identified as an independent predictor of mortality in STEMI patients with concurrent COVID-19.

**Figure 2:**
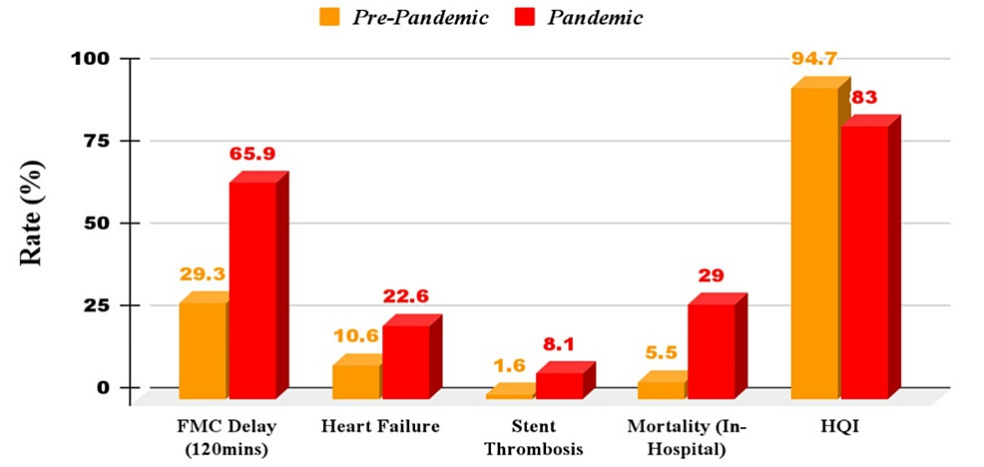
Comparison of STEMI outcomes during pre-pandemic and COVID-19 pandemic. FMC: first medical contact; HQI: health quality indicator; STEMI: ST-elevation myocardial infarction Sources: [35,56]

Figure 2 shows the effect of the COVID-19 pandemic on STEMI outcomes.

The age of STEMI patients is an important factor in determining the appropriate management of their condition, especially during the COVID-19 pandemic. Research indicates that younger STEMI patients without COVID-19 were more likely to receive invasive management than their older counterparts (65% vs. 54%) [58]. On the other hand, STEMI patients with COVID-19 were less likely to receive invasive management (54% vs. 63%) [58]. Interestingly, a study found that the incidence of acute MI decreased predominantly among women aged 70 years or older during the COVID-19 pandemic, highlighting potential disparities in healthcare access and utilization among different age groups during the pandemic [17]. 

The impact of the COVID-19 pandemic was worst on patients with comorbidities like cardiovascular diseases (CVDs) with a mortality rate of over 10% of total patients with CVDs such as STEMI, according to reports from China [55]. This mortality rate increases with age ranging from less than 1% for patients under 50 years of age to more than 15% for people aged 80 years or more [59].

Effect of COVID-19 vaccination on STEMI

Due to the lack of established clinical interventions for treating COVID-19, attention has been directed towards the development of vaccines as a preventive measure [60]. Consequently, the World Health Organization (WHO) has endorsed the safety and effectiveness of various vaccines, such as AstraZeneca/Oxford, Johnson & Johnson/Janssen, Moderna, Pfizer/BioNTech, Sinopharm, Sinovac, and Bharat Biotech BBV152 Covaxin [61].

Vaccines designed to counter SARS-CoV-2 employ various mechanisms to trigger immune responses. These methods include RNA and DNA vaccines that utilize genetic engineering techniques to generate specific proteins, vector vaccines that use harmless viruses as carriers for genetic material delivery, genetically modified virus vaccines altering viruses to preserve antigenicity while eliminating disease-causing ability, and protein-based vaccines using harmless fragments or shells to mimic viral properties [62,63]. These diverse approaches aim to elicit immune responses and provide immunity against COVID-19. These vaccine strategies leverage distinct mechanisms to elicit immune responses against SARS-CoV-2, with the goal of providing effective protection against COVID-19 [62].

Studies have shown that individuals may experience side effects following COVID-19 vaccination. These side effects encompass a spectrum of symptoms, including fatigue, headache, muscle and joint pain, chills, fever, generalized body discomfort, and localized reactions at the injection site. Typically, these reactions are mild and transient, resolving within a few days. Consequently, the occurrence of such side effects reflects a normal response by the body's immune system as it develops protection against the introduced virus in the vaccine [64]. The reactivity of different COVID-19 vaccines is influenced by various factors, including age, gender, psychological and physical stressors, obesity, pre-existing immunity, and vaccine characteristics such as route, site, method of administration, composition, type of antigen, combination of antigens, and dosage [65].

Furthermore, various adverse cardiovascular effects have also been documented following COVID-19 vaccination, predominantly including myocarditis, pericarditis, and thrombotic events. Additionally, occurrences like hypertension, ACS, stress cardiomyopathy, MI, arrhythmias, and cardiac arrest have been observed. Several studies, particularly case reports, have discussed the occurrence of MI following COVID-19 vaccination. However, these studies have not established a direct causal link between MI and the COVID-19 vaccine. A documented case report in India illustrates the occurrence of STEMI following COVID-19 vaccination [66]. While the correlation between these infrequent events and vaccination remains uncertain, their manifestation post-vaccination in healthy individuals without other apparent causes could suggest a potential association [67]. Potential mechanisms contributing to MI post-vaccination encompass Kounis syndrome inducing MI through allergic vasospasm or stent occlusion infiltrated by eosinophils or mast cells; vaccine-induced thrombotic thrombocytopenia characterized by abnormal blood clot formation and low platelet levels; ACS following influenza vaccine administration due to shared excipients like polysorbate 80 with COVID-19 vaccines; demand-supply mismatch ischemia triggered by vaccination-related stress, especially in elderly individuals with comorbidities; and myocarditis that can rarely occur post-vaccination, potentially leading to MI [68-70]. It has been observed that MI following the administration of COVID-19 vaccination occurred most frequently in males with a mean age of 63.45 years [69]. Moreover, AstraZeneca vaccinations were associated with a higher incidence of coronary artery disease, particularly STEMI, primarily observed after the initial vaccine dose [71]. In contrast, a study comparing rates of acute MI after COVID-19 infection and among vaccinated populations demonstrated much higher acute MI rates in the unvaccinated compared to those not vaccinated with COVID-19 vaccine. Post-vaccination, acute MI rates were significantly lower than those associated with COVID-19 infection in other countries [72]. Some studies exhibit conflicting outcomes, with some suggesting that COVID-19 vaccination might decrease the risk of cardiovascular events like myocarditis and MI, while others indicate an increased risk [73]. Further investigation into the relationship between COVID-19 vaccinations and MI, considering various demographic and health-related factors, is essential to comprehensively understand the potential risks and benefits associated with vaccination.

## Conclusions

The COVID-19 pandemic has significantly impacted the healthcare system, including the management of STEMI. Changes in hospitalization rates, treatment strategies, and disease presentations have posed considerable challenges to health outcomes. The pandemic's broader effects, such as transportation limitations and the fear of exposure, have also played a role. Hospitalization rates, while providing a general indication, may not accurately reflect the true incidence of STEMI during the pandemic. Conflicting study results suggest that COVID-19 vaccination could either increase or decrease the risk of post-vaccination MI and there is a noticeable gender disparity in reported cases. The reasons for these variations remain unclear, highlighting the need for further research to elucidate the relationship between COVID-19 vaccination and MI outcomes.
